# Hs-CRP/HDL-C ratio as a predictive inflammatory-lipid marker for sarcopenia: evidence from NHANES 2015–2018

**DOI:** 10.3389/fimmu.2025.1600421

**Published:** 2025-06-19

**Authors:** Xin-Yan Guo, Liang-Wen Li

**Affiliations:** ^1^ Department of Orthopaedics, Tiantai County Chinese Medicine Hospital, Taizhou, Zhejiang, China; ^2^ Department of Orthopaedics, The Second People’s Hospital of Yuhang District, Hangzhou, Zhejiang, China

**Keywords:** Hs-CRP/HDL-C ratio, inflammation, lipid metabolism, NHANES, cross-sectional study

## Abstract

**Objective:**

This study aimed to investigate the association between the high-sensitivity C-reactive protein/high-density lipoprotein cholesterol (Hs-CRP/HDL-C) ratio and sarcopenia among U.S. population by using composite marker integrating systemic inflammation and lipid metabolism.

**Method:**

This study included participants from the 2015–2018 of the National Health and Nutrition Examination Survey (NHANES). Sarcopenia was diagnosed based on the skeletal muscle index (SMI) by using dual-energy X-ray absorptiometry (DXA) technology. The Hs-CRP/HDL-C ratio was derived from laboratory examinations, was categorized into quartiles. Multivariate logistic regression models, adjusted for demographic and clinical covariates, were employed to assess the association between the Hs-CRP/HDL-C ratio and sarcopenia. Subgroup analysis and restricted cubic splines (RCS) were used to examine potential nonlinear relationships and threshold effects. Receiver operating characteristic (ROC) curve was used to evaluate the performance of the Hs-CRP/HDL-C ratio, Hs-CRP, and HDL-C indicators in predicting sarcopenia.

**Results:**

This study finally recorded 4,152 participants from NHANES from 2015–2018 for analysis. Elevated Hs-CRP/HDL-C ratios were significantly associated with an increased risk of sarcopenia, with a dose-response relationship observed across quartiles (*p* for trend <0.05). The fully adjusted model revealed that each unit increase in the Hs-CRP/HDL-C ratio corresponded to a 6% higher risk of sarcopenia (OR=1.06, 95% CI: 1.04–1.08, *p*=0.004). Participants in the highest quartile (≥2.75) of the Hs-CRP/HDL-C ratio had a 122% higher risk of sarcopenia compared to those in the lowest quartile (*p*=0.005). Subgroup analyses showed a stronger association in adults aged ≥40 years, with notable differences across races, especially among non-Hispanic Whites and other race. RCS model identified a non-linear association, with a threshold effect at a ratio of 0.86. The combined inflammatory and lipid markers Hs CRP/HDL-C showed stronger predictive performance (AUC: 0.685, 95% CI 0.666-0.703).

**Conclusion:**

The Hs-CRP/HDL-C ratio can serve as a practical indicator to identify individuals at higher risk of sarcopenia, capturing the combined effects of chronic inflammation and lipid metabolism issues. This ratio may be useful for early detection and prevention efforts targeting sarcopenia. However, more prospective research is needed to confirm these findings.﻿

## Introduction

Sarcopenia was initially conceptualized by Rosenberg et al. in 1989 as an age-related decline in skeletal muscle mass (1), and now is recognized as a multifaceted syndrome characterized by the progressive loss of muscle mass and strength associated with aging (2). This condition results in diminished physical power, impaired metabolic efficiency, and reduced aerobic capacity, profoundly compromising functional ability (3). Against the backdrop of global demographic aging, sarcopenia has emerged as a significant public health challenge, affecting 5%-10% of the worldwide population and contributing substantially to escalating healthcare expenditures (4).

Chronic inflammation and lipid metabolism disorders are key mechanisms in the development of sarcopenia (5, 6). Persistent inflammation activates molecular pathways that disrupt protein synthesis and degradation balance, accelerating muscle loss (7, 36). These effects are attributed to the catabolic impact of inflammatory markers and impaired muscle regeneration (37). Elevated circulating cytokines are also associated with higher sarcopenia prevalence, underscoring the role of systemic inflammation (38). High-sensitivity C-reactive protein (Hs-CRP) and CRP are widely used markers of systemic inflammation (8). Meta-analyses have shown that elevated CRP and Hs-CRP levels are significantly associated with reduced muscle strength and mass, supporting a link between systemic inflammation and sarcopenia (11, 30). A population-based cross-sectional study from Korea demonstrated that higher serum Hs-CRP levels were independently and inversely associated with handgrip strength in men, particularly in older males (38).

Dyslipidemia contributes to ectopic lipid deposition within and surrounding muscle fibers, thereby compromising both muscle quality and mass (9). These detrimental effects may be driven by mitochondrial dysfunction, oxidative stress, and elevated inflammatory activity (9). As a key indicator of lipid metabolism, high-density lipoprotein cholesterol (HDL-C) plays an important role in metabolic homeostasis. Recent studies have reported that lipid ratios such as non-HDL/HDL-C and LDL/HDL-C are positively associated with sarcopenia, with inflammatory biomarkers acting as partial mediators of these associations (10). Furthermore, meta-analyses have identified a significant link between elevated HDL-C levels and increased sarcopenia risk, notably among older adults and individuals with diabetes (12–14).

More recently, the Hs-CRP/HDL-C ratio has emerged as a novel biomarker that integrates two critical pathological processes-inflammation and lipid dysregulation. This ratio has shown superior predictive value compared to either marker alone in various chronic conditions, including cardiovascular disease, metabolic dysfunction-associated fatty liver disease (MAFLD), liver fibrosis, and all-cause mortality (13–15). The rationale for using this ratio lies in its ability to reflect a composite inflammatory-metabolic state, which is particularly relevant in aging-related disorders such as sarcopenia. However, its role in predicting sarcopenia remains blank.

Therefore, this study aimed to investigate the association between the Hs-CRP/HDL-C ratio and sarcopenia in a nationally representative sample of U.S. adults. Specifically, we sought to (1) assess whether this ratio is independently associated with sarcopenia prevalence, and (2) compare its predictive performance with Hs-CRP and HDL-C individually. This research may help determine whether Hs-CRP/HDL-C serves as a novel integrative biomarker for identifying individuals at elevated risk, thereby supporting early detection and targeted intervention strategies.

## Methods

### Study design

This study was a cross-sectional design and was reported in accordance with the Strengthening the Reporting of Observational Studies in Epidemiology (STROBE) guidelines.

The NHANES is administered by the National Center for Health Statistics (NCHS) and is a continuous study designed to evaluate the health and nutritional status of the U.S. population. This survey systematically collects a wide range of data, including demographic information, physical measurements, laboratory test results, and dietary intake records. To ensure representativeness, NHANES employs a complex, stratified, multistage probability cluster sampling method. Detailed protocols, data collection procedures, and access to the data are documented on the official website (https://www.cdc.gov/nchs/nhanes). Additional analytical guidelines are available in the NHANES process manual (https://wwwn.cdc.gov/nchs/nhanes/analyticguidelines.aspx). All participants provided written informed consent before their involvement in the study.

### Inclusion and exclusion criteria

Participants were included if they were (1) aged 20 years or older; (2) with complete data on Hs-CRP, HDL-C, dual-energy X-ray absorptiometry (DXA)-based appendicular skeletal muscle mass (ASM), height and weight. Exclusion criteria were (1) aged under 20 years old; (2) missing values for Hs-CRP, HDL-C, ASM, height or weight; (3) participants with Hs-CRP ≥10 mg/L, or those who reported a diagnosis of cancer, chronic respiratory conditions, or coronary heart disease in the medical questionnaire section.

### Population selection

This study initially included 19,225 participants from two cycles of NHANES survey from 2015 to 2018. After excluding individuals under 20 years old (N=7,937), missing HDL-C data (N=1,194), incomplete Hs-CRP records (N=58), absent limb measurement data (N=5,250), and missing BMI (Body mass index) data (N=15), a total of 4,771 participants aged 20–59 years remained.

Further exclusions were made for participants with Hs-CRP ≥10 mg/L (N=378), coronary heart disease (N=40), lung disease (N=59), and malignant tumors (N=142) to minimize confounding factors that could influence systemic inflammation.

**Figure 1:**
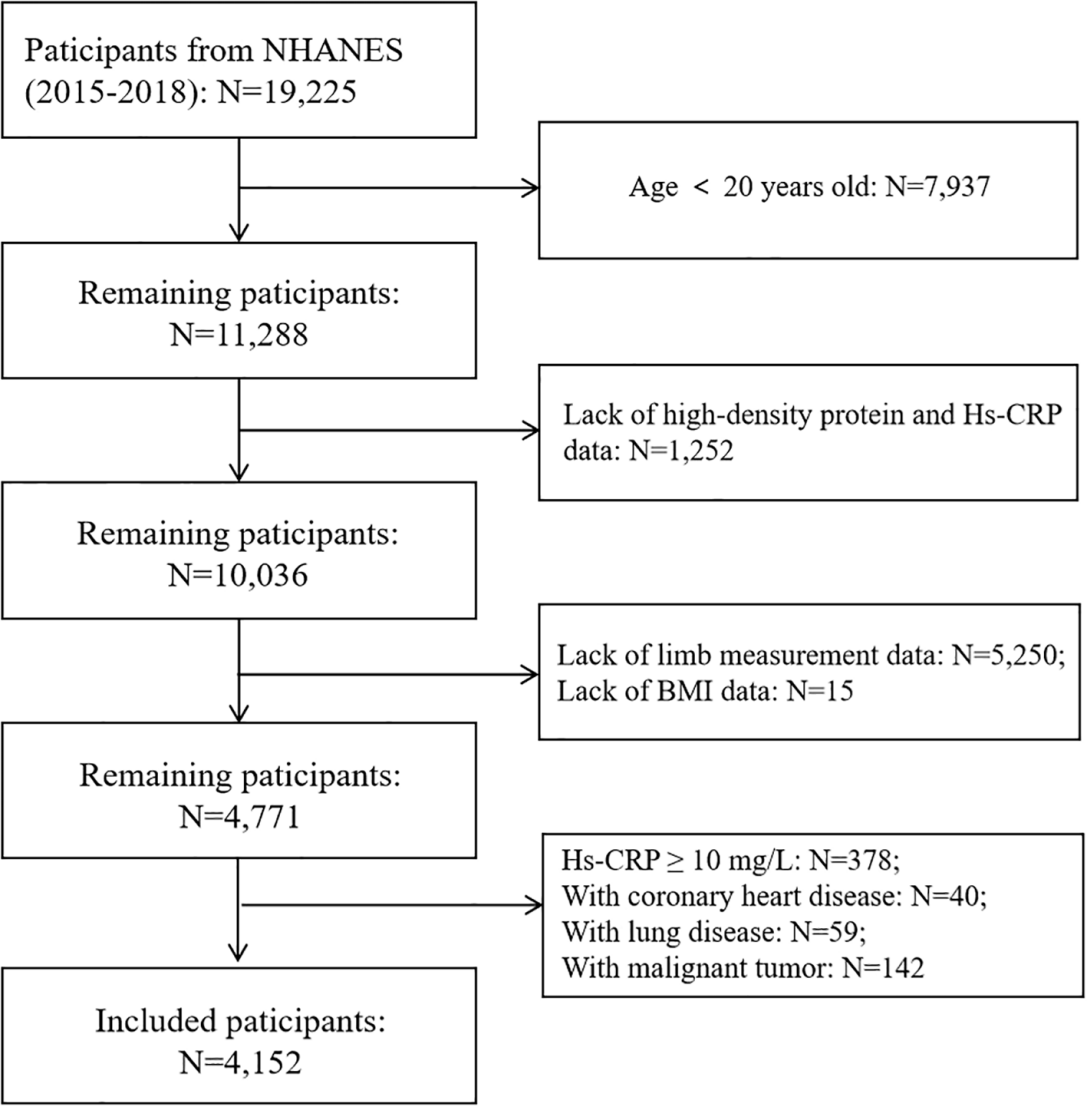
The flowchart of the included participants from NHANES 2015-2018.

The final analytic cohort included 4,152 participants, among whom 364 were classified as having sarcopenia, accounting for 8.77% of the study population. The comprehensive participant selection process is depicted in **Figure 1**.

### Data extraction

We included data from the database based on research needs. Demographic information includes race, gender, and age. Anthropometric measurements consist of height, weight, and BMI. Laboratory test results cover triglycerides, total cholesterol, low-density lipoprotein (LDL-C), HDL-C, and Hs-CRP. Additionally, self-questionnaire responses provide details on diabetes status, hypertension status, marital status, and poverty income ratio (PIR). All data were systematically gathered by professionally trained health technicians at the Mobile Examination Center using standardized procedures.

### Definition of sarcopenia

ASM is a key indicator of overall muscle health and typically measured by evaluating muscle mass in the limbs. For the NHANES study, ASM was quantified using DXA technology, based from Hologic system based in Bedford, Massachusetts. BMI was derived by dividing body weight in kilograms by the square of height in meters. The skeletal muscle index (SMI), a standardized metric, was calculated as the ratio of total ASM to BMI. Sarcopenia, a condition characterized by muscle loss, was identified using validated diagnostic thresholds: an SMI < 0.789 for males and < 0.512 for females (18).

### Calculation of Hs-CRP/HDL-C

Hs-CRP was measured using the Beckman Coulter UniCel DxC 600 Synchron and DxC 660i Synchron Access chemistry analyzers. HDL-C was determined through blood biochemistry tests in the laboratory. The ratio of Hs-CRP and HDL-C as the core indicator of this study is used to further analyze.

### Covariables

The selection of covariates was based on prior research (15–17). Data on covariates, including age, gender, race/ethnicity, PIR, triglycerides, LDL-C, hypertension status, diabetes status, and marital status, were collected for analysis. Gender, race, hypertension, diabetes, and marital status were treated as categorical variables. Marital status was grouped into two categories: married/living with a partner and others. BMI was categorized into four groups: under 18.5, 18.5-24.9, 25-29.9, and over 30. PIR was categorized into three groups: under 1.5, 1.5-3, and over 3. Race/ethnicity was classified as Non-Hispanic White, Non-Hispanic Black, Mexican American, Other Hispanic, or Other Race/Ethnicity, based on self-reported questionnaire responses. Hypertension status was dichotomized as “Yes” or “No,” while diabetes status was categorized as “Yes”, “No”, or “Borderline.” All other covariates are treated as continuous variables.

### Statistical analysis

Descriptive statistics were employed to summarize the characteristics of the study population. Continuous variables are presented as mean ± standard deviation (SD), while frequencies or percentages are used for categorical variables. Missing data were handled using multiple imputation methods. The Hs-CRP/HDL-C ratio was categorized into quartiles. The Shapiro-Wilk test was conducted to assess the normality of the distribution of continuous variables. Nonnormally distributed variables are presented as medians (interquartile ranges, IQRs), whereas categorical variables are presented as proportions. The Mann-Whitney U test or X*^2^* test was applied for group comparisons.

We used weighted logistic regression models that accounted for the complex survey design of NHANES to examine the association between the Hs-CRP/HDL-C ratio and sarcopenia. In Model 1, no covariates were adjusted. Model 2 controlled for age, gender, and race, while Model 3 further adjusted for BMI, triglyceride levels, LDL-C, diabetes status, hypertension status, and the poverty-to-income ratio (PIR). Subgroup analyses were performed using a hierarchical multivariable logistic regression model with stratification based on gender, race and ethnicity (Mexican American, other Hispanic, non-Hispanic White, non-Hispanic Black, and other races), marital status (married/living with partner vs. other), PIR categories (<1.5, 1.5-3.5, and >3.5), and the presence of hypertension (yes/no) and diabetes (yes/no/borderline).

To evaluate potential nonlinear associations between the Hs-CRP/HDL-C ratio and sarcopenia, restricted cubic spline (RCS) regression was applied, using four knots to flexibly model the relationship through smooth, piecewise cubic functions (39).When nonlinearity was observed, a two-piecewise linear regression model was applied to identify potential threshold effects. The optimal inflection point (K) was determined using a recursive algorithm that tested all possible values within the specified range and selected the one that maximized the model log-likelihood (40, 41). The algorithm starts with random initialization and then uses smoothing steps to identify inflection points. The predictive ability of Hs-CRP, HDL-C, and the Hs-CRP/HDL-C ratio was assessed by plotting receiver operating characteristic (ROC) curves and determining area under the curve (AUC) values.

All statistical analyses were performed using R version 4.1 and Empower software (www.Empowerstats.com; X&Y Solutions, Inc., Boston, Massachusetts). A *p*-value <0.05 was considered statistically significant.

## Results

### Baseline characteristics of study participants

This study analyzed a cohort of 4,152 participants derived from the NHANES 2015–2018 cycles. **Table 1** presents the baseline characteristics of the study participants, stratified by quartiles of the Hs-CRP/HDL-C ratio. The mean age of the participants was 38.94 ± 11.42 years, with males taking 50.36% of the cohort. 35.31% of participants had a BMI > 30, 29.17% identified as Non-Hispanic White. A significant proportion (61.54%) reported being married or living with a partner, and 34.01% exhibited a PIR < 1.5.

**Table 1:** Baseline of the participants included from NHANES 2015-2018, Hs-CRP/HDL-C is represented in quartiles.

Variables	Overall	Q1 (<0.47)	Q2 (0.47 to 1.17)	Q3 (1.17 to 2.75)	Q4 (≥2.75)	P-value
Total Number	4,152	1,038	1,038	1,038	1,038	
Number of Sarcopenia	364	22	80	107	155	<0.001
Proportion of Sarcopenia (%)	8.77	2.11	7.71	10.31	14.93	<0.001
Gender						0.032
Male	2091 (50.36%)	507 (48.84%)	549 (52.89%)	543 (52.31%)	492 (47.40%)	
Female	2061 (49.64%)	531 (51.16%)	489 (47.11%)	495 (47.69%)	546 (52.60%)	
Hs-CRP/HDL-C	1.95±2.08	0.25±0.13	0.77±0.20	1.85±0.46	4.92±1.97	<0.001
Age (years old)	38.94±11.42	36.57±11.57	39.26±11.54	39.80±11.25	40.12±10.96	<0.001
Height (cm)	166.81±9.2	167.44±9.22	166.73±9.38	166.82±9.75	166.27±9.72	0.045
Weight (kg)	79.52±19.76	67.27±14.04	76.11±15.45	82.81±18.06	91.87±21.81	<0.001
BMI (kg/m^2^)	28.49±6.3	23.87±3.90	27.32±4.79	29.69±5.66	33.09±6.72	<0.001
BMI group						<0.001
<18.5	76 (1.83%)	53 (5.11%)	11 (1.06%)	5 (0.48%)	7 (0.67%)	
18.5 to 24.9	1255 (30.23%)	638 (61.46%)	321 (30.93%)	208 (20.04%)	88 (8.48%)	
25 to 29.9	1355 (32.63%)	271 (26.11%)	450 (43.35%)	370 (35.65%)	264 (25.43%)	
>30	1466 (35.31%)	76 (7.32%)	256 (24.66%)	455 (43.83%)	679 (65.41%)	
Race						<0.001
Mexican American	737 (17.76%)	104 (10.02%)	198 (19.08%)	208 (20.04%)	227 (21.87%)	
Other hispanic	495 (11.92%)	95 (9.15%)	123 (11.85%)	131 (12.62%)	146 (14.07%)	
Non-hispanic White	1,211 (29.17%)	312 (30.06%)	296 (28.52%)	302 (29.09%)	301 (29.00%)	
Non-hispanic Black	817 (19.68%)	217 (20.91%)	181 (17.44%)	212 (20.42%)	207 (19.94%)	
Other race	892 (21.48%)	310 (29.87%)	240 (23.12%)	185 (17.82%)	157 (15.13%)	
Martial status						0.009
Married or lived with partner	2,555 (61.54%)	594 (57.23%)	650 (62.62%)	646 (62.24%)	665 (64.07%)	
Other status	1,597 (38.46%)	444 (42.77%)	388 (37.38%)	392 (37.77%)	373 (35.93%)	
PIR						<0.001
<1.5	1,412 (34.01%)	305 (29.38%)	340 (32.76%)	358 (34.49%)	409 (39.40%)	
1.5 to 3.5	1,450 (34.92%)	356 (34.30%)	356 (34.30%)	359 (34.59%)	379 (36.51%)	
>3.5	1,290 (31.07%)	377 (36.32%)	342 (32.95%)	321 (30.93%)	250 (24.09%)	
Hypertension status						<0.001
Yes	875 (21.07%)	128 (12.33%)	196 (18.88%)	241 (23.22%)	310 (29.87%)	
No	3,277 (78.93%)	910 (87.67%)	842 (81.12%)	797 (76.78%)	728 (70.14%)	
Diabetes status						<0.001
Yes	268 (6.45%)	32 (3.08%)	44 (4.24%)	75 (7.23%)	117 (11.27%)	
No	3,815 (91.88%)	997 (96.05%)	979 (94.32%)	941 (90.66%)	898 (86.51%)	
Bordline	69 (1.66%)	9 (0.87%)	15 (1.45%)	22 (2.12%)	23 (2.22%)	
Triglyceride (mmol/L)	1.32±1.42	0.89±0.61	1.30±1.16	1.40±1.39	1.72±2.03	<0.001
LDL-C (mmol/L)	2.96±0.89	2.72±0.85	3.01±0.87	3.05±0.89	3.06±0.91	<0.001
HDL-C (mmol/L)	1.37±0.41	1.66±0.44	1.38±0.37	1.28±0.35	1.15±0.30	<0.001
Hs-CRP (mg/L)	2.31±2.20	0.40±0.22	1.06±0.37	2.36±0.84	5.43±1.91	<0.001

BMI, body mass index; PIR, poverty income ratio; LDL-C, low-density lipoprotein cholesterol; HDL-C, high-density lipoprotein cholesterol, High-sensitivity C-reactive Protein.

Comparative analysis revealed that participants in the higher quartiles of the hs-CRP/HDL-C ratio were more likely to exhibit a higher BMI, lower PIR (<1.5), and an increased risk of hypertension and diabetes, relative to those in the lower quartiles. Their Hs-CRP levels were significantly elevated.

### The association between Hs-CRP/HDL-C and sarcopenia

**Table 2** presents the relationship between the Hs-CRP/HDL-C ratio and sarcopenia. The results showed that higher Hs-CRP/HDL-C levels were significantly associated with increased odds of sarcopenia. This positive association remained consistent after adjusting for all relevant covariates in the fully adjusted model (OR=1.06; 95% confidence interval [CI]: 1.04-1.08; *p*=0.004). Each unit increase in the Hs-CRP/HDL-C ratio was associated with a 6% higher odds of sarcopenia.

**Table 2:** Relationship between Hs-CRP/HDL-C and sarcopenia in different models.

Variables	TN	NoS	OR (95% CI), *p*-value		
			Model 1	Model 2	Model 3
Hs-CRP/HDL-C	4,152	364	1.10 (1.04-1.15), <0.001	1.09 (1.04-1.15), <0.001	1.06 (1.04-1.08), 0.004
Quartile of Hs-CRP/HDL-C					
Q1 (<0.47)	1,038	22	Reference	Reference	Reference
Q2 (0.47 to 1.17)	1,038	80	2.45 (1.50-4.02, <0.001	1.99 (1.20-3.31), 0.008	1.90 (1.13-3.19), 0.016
Q3 (1.17 to 2.75)	1,038	107	2.77 (1.69-4.52), <0.001	2.39 (1.44-3.97), <0.001	2.14 (1.26-3.63), 0.005
Q4 (≥2.75)	1,038	155	3.53 (2.16-5.76), <0.001	3.02 (1.82-5.01), <0.001	2.22 (1.28-3.85), 0.005
*p* for trend	/	/	<0.001	<0.001	0.019

Model 1: adjusted for no covariates.

Model 2: adjusted for gender, age, race.

Model 3: adjusted for gender, age, race, BMI, triglyceride, LDL-C, diabetes status, hypertension status and PIR.

BMI, Body mass index; CI, Confidence interval; LDL-C, Low-density lipoprotein; TN, Total Number; NoS, Number of Sarcopenia; OR, Odds ratio; PIR, Poverty to income ratio.

Participants in the highest quartile exhibited a 122% greater risk (OR: 2.22, 95% CI 1.28-3.85) of sarcopenia compared to those in the lowest quartile, with statistical significance (*p*=0.005). Across all models, the odds of sarcopenia increased progressively with higher Hs-CRP/HDL-C quartiles. The *p* for trend was statistically significant (*p*<0.05) in all models, indicating a dose-response relationship between the Hs-CRP/HDL-C ratio and the likelihood of sarcopenia.

### Subgroup analysis

**Figure 2:**
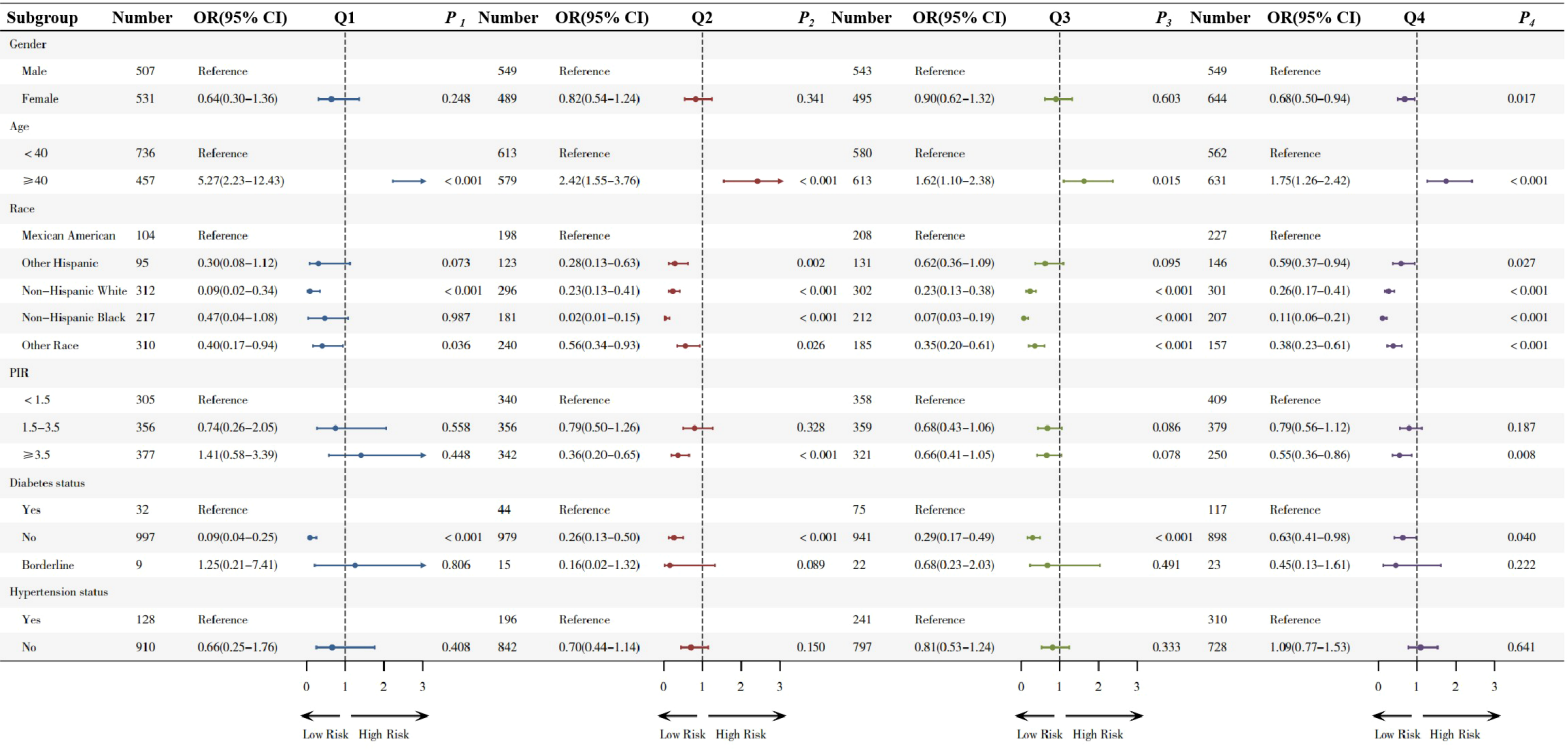
Subgroup analysis of the association between Hs-CRP/HDL-C quartiles and sarcopenia.

Subgroup analysis in quartiles are shown in **Figure 2**. Compared to males, females showed a protective effect only in the highest quartile (OR 0.68,95% CI 0.50-0.94, *p*=0.017), while no significant differences were observed in the other three quartiles.

In the age-based subgroup, adults aged ≥40 years exhibited significantly elevated odds of sarcopenia across all higher quartiles compared to less than 40 year old (Q1: OR=5.27; Q2: OR=2.42; Q3: OR=1.62 and Q4: OR=1.75).

In the diabetes subgroup analysis, non-diabetic individuals exhibited a decreasing risk of sarcopenia across all quartiles, with ORs of 0.09 (Q1), 0.26 (Q2), 0.29 (Q3), and 0.63 (Q4), indicating a consistent protective effect. No significant associations were observed in the hypertension subgroup (with or without), with all p-values > 0.05.

### RCS model and threshold effect

**Figure 3:**
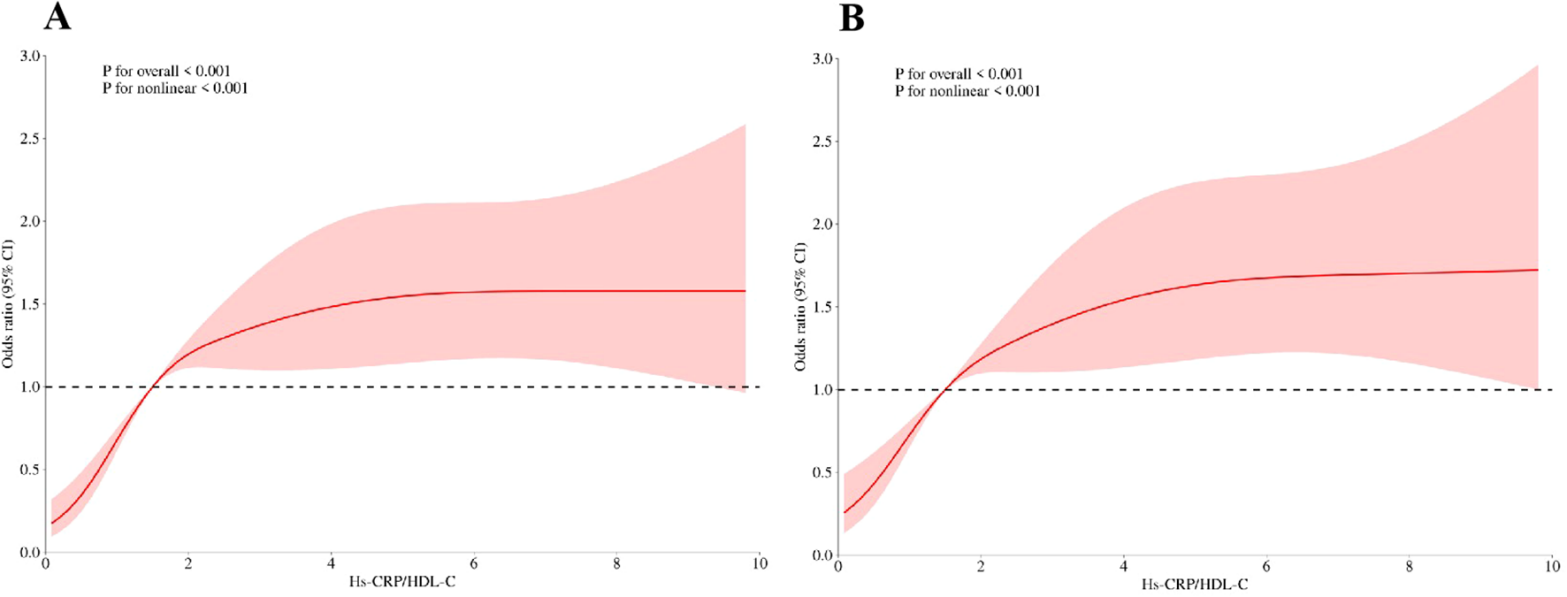
**(A)**The risk between Hs-CRP/HDL-C and sarcopenia under RCS model, with no covariables adjusted; **(B)** The risk between Hs-CRP/HDL-C and sarcopenia under RCS model, with adjustment of age, BMI, race, LDL-C, triglycerides, martial status, PIR, hypertension status and diabetes status.

The RCS model was shown in **Figure 3**. The RCS model revealed a non-linear correlation between the Hs-CRP/HDL-C ratio and sarcopenia (*p* for non-linearity <0.001, **Figure 3A**), with no covariables adjusted. After adjusting for covariates, non-linear correlation still exists (**Figure 3B**).

Threshold effects are summarized in **Table 3**. The estimated turning point (K) of 0.86 was identified using a threshold effect model. On the left side of the threshold, the Hs-CRP/HDL-C ratio was significantly associated with increased odds of sarcopenia (OR=4.34, 95% CI: 1.98-9.52, *p*<0.001). However, on the right side of the threshold, no statistically significant association was observed (OR=0.99, 95% CI: 0.93-1.05, *p*=0.656). The log-likelihood ratio test supported the presence of a threshold effect (*p*<0.001).

**Table 3:** The threshold effect analysis of the Hs-CRP/HDL-C on sarcopenia risk among adults in NHANES 2013–2018.

EModel	OR (95% CI)	*P*-value
Model 1		
The standard linear model	1.18 (1.13 ~ 1.24)	<0.001
Model 2		
Turning point (K)	0.86	
Hs-CRP/HDL-C≤0.86	4.34 (1.98 ~ 9.52)	<0.001
Hs-CRP/HDL-C>0.86	0.99 (0.93 ~ 1.05)	0.656
Log likelihood ratio test		<0.001

Model 1 was a standard linear model. Model 2 was a piecewise model, adjusted factors such as gender, age, BMI, marital status, diabetes status and hypertension status. Log likelihood ratio test was used to compare two models.

BMI, body mass index.

### ROC curves

**Table 4**; **Figure 4** provided the AUC levels and 95% confidence intervals (95% CI) for three indicators for sarcopenia in US adults. Compared to individual indicators, the combined inflammatory and lipid markers Hs-CRP/HDL-C showed stronger predictive performance (AUC: 0.685, 95% CI 0.666-0.703).

**Table 4:** Results of ROC analysis of three test variables.

Test Variables	AUC	95% CI lower	95% CI upper	Specificity	Sensitivity
Hs-CRP/HDL-C	0.6849	0.6664	0.7031	0.6244	0.8379
Hs-CRP	0.6351	0.6072	0.6630	0.6013	0.6648
HDL-C	0.6197	0.5953	0.6440	0.5327	0.6621

Hs-CRP, High-sensitivity C-reactive protein; HDL-C, High-density lipoprotein cholesterol.

**Figure 4:**
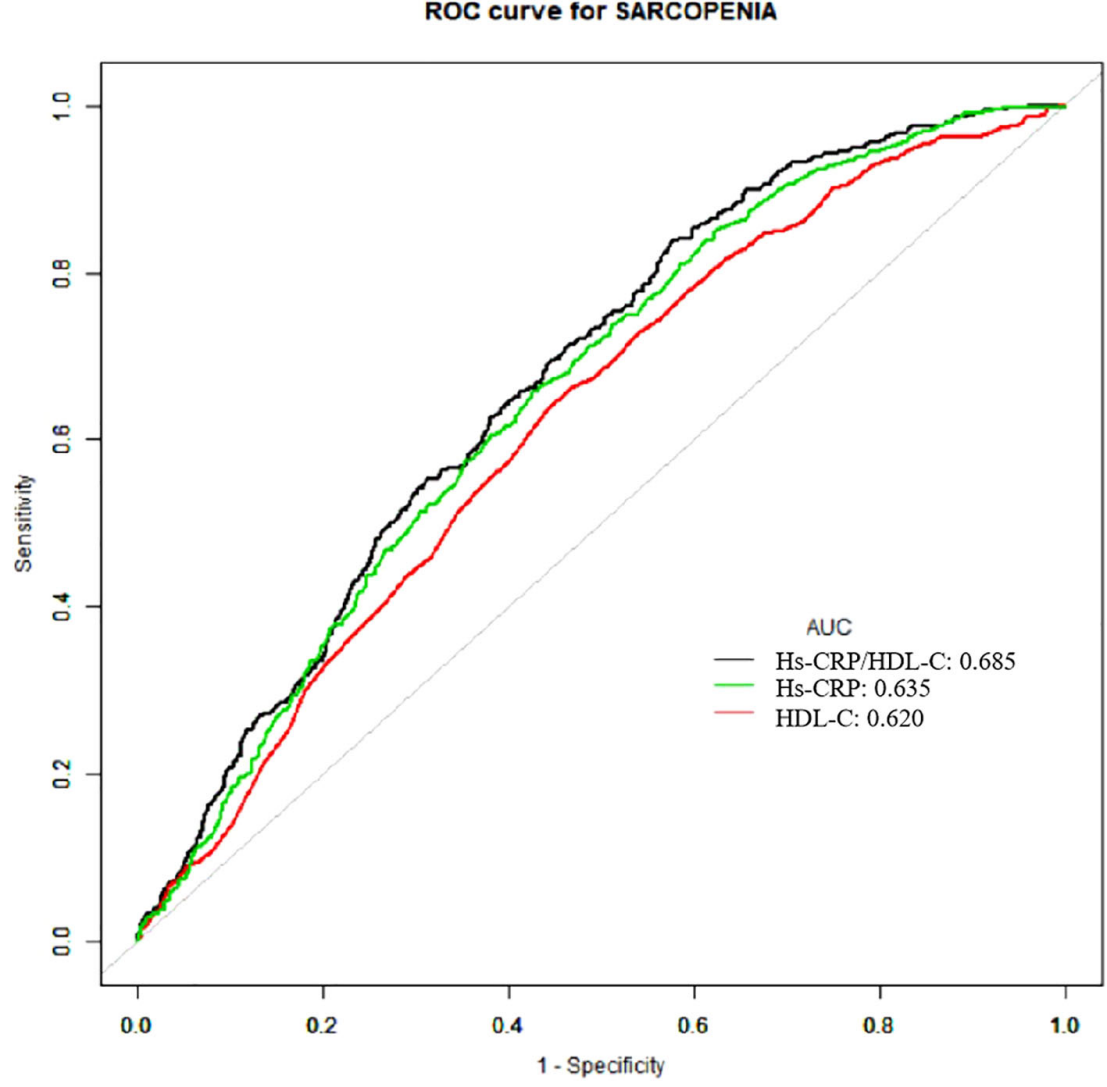
ROC curves of three indicators for predicting sarcopenia.

## Discussion

This study is the first to explore the association between the Hs-CRP/HDL-C ratio and sarcopenia using data from the NHANES survey. Among the 4,152 adults included in the analysis, 364 were identified with sarcopenia, corresponding to a prevalence of 8.77%. When stratified by Hs-CRP/HDL-C quartiles, the proportion of sarcopenia increased progressively from 2.11% in Q1 to 14.93% in Q4. This graded pattern supports a potential dose-dependent relationship, indicating that a higher inflammatory-to-lipid ratio may be linked to a greater likelihood of sarcopenia.

In addition, the association between the Hs-CRP/HDL-C ratio and sarcopenia followed a nonlinear trend. The ratio demonstrated better predictive value than either marker alone, as reflected by a higher AUC in ROC analysis (AUC=0.685). These findings suggest that the Hs-CRP/HDL-C ratio may offer clinical utility as an integrated marker for the early identification of individuals at elevated risk for sarcopenia.

Sarcopenia is a complex metabolic disorder characterized by impaired protein turnover, frequently associated with aging and chronic low-grade inflammation (19, 20). Elevated levels of pro-inflammatory cytokines such as TNF-α and IL-6 have been linked to reduced skeletal muscle mass and increased fat infiltration in older adults (21–23, 26). Aging also promotes an imbalance between M1 and M2 macrophages in muscle tissue, which may amplify catabolic processes (24, 25). These mechanisms may explain why the association between Hs-CRP/HDL-C and sarcopenia was particularly evident in individuals aged 40 and above. In parallel, obesity-induced lipid overload activates endoplasmic reticulum stress and pro-inflammatory signaling in adipose tissue, particularly in areas surrounding skeletal muscle. This promotes local inflammation, immune cell infiltration, and tissue remodeling, ultimately contributing to sarcopenia (33–35).

CRP is a well-established marker of systemic inflammation, and Hs-CRP offers greater sensitivity in detecting subtle clinical changes (27, 28). Previous meta-analyses have shown that elevated levels of both CRP and Hs-CRP are associated with reduced muscle strength (30). Although HDL-C generally has anti-inflammatory properties, it may become dysfunctional and even pro-inflammatory under pathological conditions such as diabetes (31, 32). These markers are involved in key biological pathways, including oxidative stress, lipid imbalance, and immune activation (15–17), particularly in individuals with diabetes. Diabetes contributes to chronic low-grade inflammation, which accelerates muscle degradation, an effect also observed in our study (29). In subgroup analyses, participants without diabetes exhibited a more pronounced protective association between the Hs-CRP/HDL-C ratio and sarcopenia, with odds ratios ranging from 0.09 to 0.63 across all quartiles. This association was not seen in those with diabetes or borderline diabetes. These findings suggest that an individual’s metabolic state plays a crucial role in modulating the relationship between systemic inflammation and muscle health.

The association between Hs-CRP/HDL-C and sarcopenia exhibited a nonlinear pattern. At low levels of Hs-CRP/HDL-C, the body may compensate for mild inflammation without significant impact on muscle protein turnover. In early stages (lower quartiles), HDL-C may still exert protective anti-inflammatory and antioxidant effects. As inflammation worsens (higher Hs-CRP) and HDL-C becomes dysfunctional or pro-inflammatory, this protective capacity diminishes, accelerating sarcopenic progression. Also, the ratio captures two interrelated pathophysiological pathways-inflammation and lipid metabolism—which may not act additively but synergistically. When both are abnormal, they may potentiate each other’s adverse effects on skeletal muscle, resulting in a nonlinear escalation of risk.

The Hs-CRP/HDL-C ratio offers a valuable tool for disease risk assessment and early intervention by integrating markers of both lipid metabolism and systemic inflammation. In our analysis, this composite indicator outperformed its individual components in predicting sarcopenia. ROC analysis showed an AUC of 0.685 (95% CI: 0.666-0.703), higher than that of Hs-CRP alone (AUC: 0.635) and HDL-C alone (AUC: 0.620), indicating improved sensitivity and specificity. This superior performance likely reflects the ratio’s ability to capture two interrelated pathogenic processes central to sarcopenia. Notably, similar predictive utility of the Hs-CRP/HDL-C ratio has been reported in cardiovascular disease, liver fibrosis, and metabolic dysfunction-associated fatty liver disease (MAFLD) (15–17), underscoring its broader clinical applicability.

This study highlights several strengths, including the utilization of NHANES data, which bolsters the objectivity of the findings. We also meticulously adjusted for potential confounders, enhancing the reliability and broader applicability of the results. However, some limitations should be acknowledged. The cross-sectional nature of the study hinders the ability to establish a causal link between Hs-CRP/HDL-C and sarcopenia, necessitating future prospective research with expanded sample sizes. Though multiple covariates were controlled, residual confounding from unmeasured factors cannot be entirely excluded. In addition, due to NHANES protocol limitations, individuals aged ≥60 years were not included, as DXA-based muscle mass data were only available for participants aged 20–59 years. Lastly, regional variations in sarcopenia diagnostic criteria exist. As the data originated from a U.S. based database, we aligned with the NIHS guidelines, which may affect the observed association between Hs-CRP/HDL-C and sarcopenia.

## Conclusions

Our findings indicate that the Hs-CRP/HDL-C ratio is a practical and timely biomarker for identifying individuals at risk of sarcopenia. It offers a simple and accessible approach that could potentially be used as a self-screening tool. However, the scope of this study is limited to a U.S. based population, emphasizing the need for broader validation across larger sample sizes and diverse international cohorts. Expanding the research in this way would improve the generalizability of our findings and provide more robust insights applicable to different demographic contexts.

## Data Availability

The datasets presented in this study can be found in online repositories. The names of the repository/repositories and accession number(s) can be found below: This study examined datasets that were accessible to the public. The location of this data can be accessed here: http://www.cdc.gov/nhanes.

## References

[1] Rosenberg IH. Sarcopenia: origins and clinical relevance. J Nutr. (1997) 127:990S–1S. doi: 10.1093/jn/127.5.990S 9164280

[2] Villareal DT Aguirre L Gurney AB. Aerobic or resistance exercise, or both, in dieting obese older adults. N Engl J Med. (2017) 376:1943–55. doi: 10.1056/NEJMoa1616338 PMC5552187 28514618

[3] Fielding RA Vellas B Evans WJ. Sarcopenia: an undiagnosed condition in older adults. Current consensus definition: prevalence, etiology, and consequences. International working group on sarcopenia. . J Am Med Dir Assoc. (2011) 12:249–56. doi: 10.1016/j.jamda.2011.01.003 PMC3377163 21527165

[4] Jyväkorpi SK Urtamo A Kivimäki M Strandberg TE. Macronutrient composition and sarcopenia in the oldest-old men: The Helsinki Businessmen Study (HBS). Clin Nutr. (2020) 39:3839–41. doi: 10.1016/j.clnu.2020.04.024 32376097

[5] Petermann-Rocha F Gray SR Pell JP Celis-Morales C Ho FK. Biomarkers profile of people with sarcopenia: A cross-sectional analysis from UK biobank. J Am Med Dir Assoc. (2020) 21:2017.e1–2017.e9. doi: 10.1016/j.jamda.2020.05.005 32641273

[6] Jung UJ. Sarcopenic obesity: involvement of oxidative stress and beneficial role of antioxidant flavonoids. Antioxidants (Basel). (2023) 12:1063. doi: 10.3390/antiox12051063 37237929 PMC10215274

[7] Kalinkovich A Livshits G. Sarcopenic obesity or obese sarcopenia: A cross talk between age-associated adipose tissue and skeletal muscle inflammation as a main mechanism of the pathogenesis. Ageing Res Rev. (2017) 35:200–21. doi: 10.1016/j.arr.2016.09.008 27702700

[8] Moutachakkir M Lamrani Hanchi A Baraou A Boukhira A Chellak S. Immunoanalytical characteristics of C-reactive protein and high sensitivity C-reactive protein. Ann Biol Clin (Paris). (2017) 75:225–9. doi: 10.1684/abc.2017.1232 28377336

[9] Li CW Yu K Shyh-Chang N Jiang Z Liu T Ma S. Pathogenesis of sarcopenia and the relationship with fat mass: descriptive review. J Cachexia Sarcopenia Muscle. (2022) 13:781–94. doi: 10.1002/jcsm.12901 PMC8977978 35106971

[10] Yin X Song H Chen H Yang X Zhang T. Association between lipid ratios and sarcopenia and the mediating roles of inflammatory biomarkers in a cross-sectional study from NHANES 2011-2018. Sci Rep. (2025) 15:6617. doi: 10.1038/s41598-025-90131-y 39994278 PMC11850801

[11] Tuttle CSL Thang LAN Maier AB. Markers of inflammation and their association with muscle strength and mass: A systematic review and meta-analysis. Ageing Res Rev. (2020) 64:101185. doi: 10.1016/j.arr.2020.101185 32992047

[12] Bi B Dong X Yan M Zhao Z Liu R Li S. Dyslipidemia is associated with sarcopenia of the elderly: a meta-analysis. BMC Geriatr. (2024) 24:181. doi: 10.1186/s12877-024-04761-4 38395763 PMC10885450

[13] Hua N Qin C Wu F Wang A Chen J Zhang Q. High-density lipoprotein cholesterol level and risk of muscle strength decline and sarcopenia in older adults. Clin Nutr. (2024) 43:2289–95. doi: 10.1016/j.clnu.2024.08.017 39217844

[14] Feng L Gao Q Hu K Wu M Wang Z Chen F. Prevalence and risk factors of sarcopenia in patients with diabetes: A meta-analysis. J Clin Endocrinol Metab. (2022) 107:1470–83. doi: 10.1210/clinem/dgab884 34904651

[15] Li J Ma H. Associations of the hs-CRP/HDL-C ratio with cardiovascular disease among US adults: Evidence from NHANES 2015-2018. Nutr Metab Cardiovasc Dis. (2024) 3:103814. doi: 10.1016/j.numecd.2024.103814 39794258

[16] Liang B Qiu X Huang J Lu Y Shen H Ma J. Nonlinear associations of the hs-CRP/HDL-C index with metabolic dysfunction-associated steatotic liver disease and advanced liver fibrosis in US adults: insights from NHANES 2017-2018. Sci Rep. (2025) 15:4029. doi: 10.1038/s41598-025-88685-y 39900651 PMC11791041

[17] Wang Y Wang L Zhao Z Yin S Tang X Zhang K. The predictive role of the hs-CRP/HDL-C ratio for long-term mortality in the general population: evidence from a cohort study. BMC Cardiovasc Disord. (2024) 24:758. doi: 10.1186/s12872-024-04446-1 39736563 PMC11684128

[18] Batsis JA Mackenzie TA Jones JD Lopez-Jimenez F Bartels SJJCN. Sarcopenia, sarcopenic obesity and inflammation: Results from the 1999–2004 National Health and Nutrition Examination Survey. Clin Nutr. (2016) 35:1472–83. doi: 10.1016/j.clnu.2016.03.028 PMC6432912 27091774

[19] Robinson SM Reginster JY Rizzoli R Shaw SC Kanis JA Bautmans I. Does nutrition play a role in the prevention and management of sarcopenia? Clin Nutr. (2018) 37:1121–32. doi: 10.1016/j.clnu.2017.08.016 PMC5796643 28927897

[20] Antuña E Cachán-Vega C Bermejo-Millo JC Potes Y Caballero B Vega-Naredo I. Inflammaging: implications in sarcopenia. Int J Mol Sci. (2022) 23:15039. doi: 10.3390/ijms232315039 36499366 PMC9740553

[21] Ferrucci L Corsi A Lauretani F Bandinelli S Bartali B Taub DD. The origins of age-related proinflammatory state. Blood. (2005) 105:2294–9. doi: 10.1182/blood-2004-07-2599 PMC9828256 15572589

[22] Pedersen M Bruunsgaard H Weis N Hendel HW Andreassen BU Eldrup E. Circulating levels of TNF-alpha and IL-6-relation to truncal fat mass and muscle mass in healthy elderly individuals and in patients with type-2 diabetes. Mech Ageing Dev. (2003) 124:495–502. doi: 10.1016/S0047-6374(03)00027-7 12714258

[23] Ali S Garcia JM. Sarcopenia, cachexia and aging: diagnosis, mechanisms and therapeutic options - a mini-review. Gerontology. (2014) 60:294–305. doi: 10.1159/000356760 24731978 PMC4112511

[24] Cui CY Ferrucci L. Macrophages in skeletal muscle aging. Aging (Albany NY). (2020) 12:3–4. doi: 10.18632/aging.102740 31937688 PMC6977691

[25] Cui CY Ferrucci L Gorospe M. Macrophage involvement in aging-associated skeletal muscle regeneration. Cells. (2023) 12:1214. doi: 10.3390/cells12091214 37174614 PMC10177543

[26] Song G Oh HJ Jin H Han H Lee BY. GABA prevents sarcopenia by regulation of muscle protein degradation and inflammaging in 23- to 25-month-old female mice. J Cachexia Sarcopenia Muscle. (2024) 15:2852–64. doi: 10.1002/jcsm.13646 PMC11634462 39513373

[27] Pepys MB. Hirschfield GM. C-reactive protein: Crit update J Clin Invest. (2003) 111:1805–12. doi: 10.1172/JCI18921 PMC161431 12813013

[28] Moutachakkir M Lamrani Hanchi A Baraou A Boukhira A Chellak S. Immunoanalytical characteristics of C-reactive protein and high sensitivity C-reactive protein. Ann Biol Clin (Paris). (2017) 75:225–9. doi: 10.1684/abc.2017.1232 28377336

[29] Hashimoto Y Takahashi F Okamura T Hamaguchi M Fukui M. Diet, exercise, and pharmacotherapy for sarcopenia in people with diabetes. Metabolism. (2023) 144:155585. doi: 10.1016/j.metabol.2023.155585 37156410

[30] Shokri-Mashhadi N Moradi S Heidari Z Saadat S. Association of circulating C-reactive protein and high-sensitivity C-reactive protein with components of sarcopenia: A systematic review and meta-analysis of observational studies. Exp Gerontol. (2021) 150:111330. doi: 10.1016/j.exger.2021.111330 33848566

[31] von Eckardstein A Nordestgaard BG Remaley AT Catapano AL. High-density lipoprotein revisited: biological functions and clinical relevance. Eur Heart J. (2023) 44:1394–407. doi: 10.1093/eurheartj/ehac605 PMC10119031 36337032

[32] Tall AR Yvan-Charvet L. Cholesterol, inflammation and innate immunity. Nat Rev Immunol. (2015) 15:104–16. doi: 10.1038/nri3793 PMC4669071 25614320

[33] Kolb H. Obese visceral fat tissue inflammation: from protective to detrimental? BMC Med. (2022) 20:494. doi: 10.1186/s12916-022-02672-y 36575472 PMC9795790

[34] Wang M Yang Z Zhai H. Association of high-density lipoprotein cholesterol with sarcopenia in chinese community-dwelling middle-aged and older adults: evidence from 4-year longitudinal study. Gerontology. (2024) 70:812–22. doi: 10.1159/000538980 38679016

[35] Pieńkowska J Brzeska B Kaszubowski M Kozak O Jankowska A Szurowska E. MRI assessment of ectopic fat accumulation in pancreas, liver and skeletal muscle in patients with obesity, overweight and normal BMI in correlation with the presence of central obesity and metabolic syndrome. Diabetes Metab Syndr Obes. (2019) 12:623–36. doi: 10.2147/DMSO.S194690 PMC6506015 31118724

[36] Sanchez-Ramirez DC van der Leeden M van der Esch M Gerritsen M Roorda LD Verschueren S. Association of serum C-reactive protein and erythrocyte sedimentation rate with muscle strength in patients with knee osteoarthritis. Rheumatol (Oxford). (2013) 52:727–32. doi: 10.1093/rheumatology/kes366 23275388

[37] Ferrucci L Penninx BW Volpato S Harris TB Bandeen-Roche K Balfour J. Change in muscle strength explains accelerated decline of physical function in older women with high interleukin-6 serum levels. J Am Geriatr Soc. (2002) 50:1947–54. doi: 10.1046/j.1532-5415.2002.50605.x 12473005

[38] Kim BJ Lee SH Kwak MK Isales CM Koh JM Hamrick MW. Inverse relationship between serum hsCRP concentration and hand grip strength in older adults: a nationwide population-based study. Aging (Albany NY). (2018) 10:2051–61. doi: 10.18632/aging.101529 PMC6128433 30115813

[39] Harre FE JrLee KL Pollock BG. Regression models in clinical studies: determining relationships between predictors and response. J Natl Cancer Inst. (1988) 80:1198–202. doi: 10.1093/jnci/80.15.1198 3047407

[40] Zhang Y Duan R Chen X Wei L. Blood pressure, gallstones, and age at first cholecystectomy in U.S. adults: a cross-sectional study. BMC Gastroenterol. (2025) 25:65. doi: 10.1186/s12876-025-03641-4 39920609 PMC11806608

[41] Yang X Chen Q Zhang Q Yu Z. Lipoprotein cholesterol ratios and cardiovascular disease risk in US adults: a cross-sectional study. Front Nutr. (2025) 12:1529223. doi: 10.3389/fnut.2025.1529223 40313882 PMC12043482

